# Effectiveness of an Individualized Training Based on Force-Velocity Profiling during Jumping

**DOI:** 10.3389/fphys.2016.00677

**Published:** 2017-01-09

**Authors:** Pedro Jiménez-Reyes, Pierre Samozino, Matt Brughelli, Jean-Benoît Morin

**Affiliations:** ^1^Faculty of Sport, Catholic University of San AntonioMurcia, Spain; ^2^Laboratoire Interuniversitaire de Biologie de la motricité (EA7424), University of Savoie Mont BlancLe Bourget du Lac, France; ^3^Sports Performance Research Institute New Zealand (SPRINZ), Auckland University of TechnologyAuckland, New Zealand; ^4^Université Côte d'Azur, LAMHESSNice, France

**Keywords:** jumping, ballistic training, explosive performance, resistance strength training, maximal power output

## Abstract

Ballistic performances are determined by both the maximal lower limb power output (*P*_*max*_) and their individual force-velocity (F-v) mechanical profile, especially the F-v imbalance (*FV*_*imb*_): difference between the athlete's actual and optimal profile. An optimized training should aim to increase *P*_*max*_ and/or reduce *FV*_*imb*_. The aim of this study was to test whether an individualized training program based on the individual F-v profile would decrease subjects' individual *FV*_*imb*_ and in turn improve vertical jump performance. *FVimb* was used as the reference to assign participants to different training intervention groups. Eighty four subjects were assigned to three groups: an “optimized” group divided into velocity-deficit, force-deficit, and well-balanced sub-groups based on subjects' *FV*_*imb*_, a “non-optimized” group for which the training program was not specifically based on *FV*_*imb*_ and a control group. All subjects underwent a 9-week specific resistance training program. The programs were designed to reduce *FV*_*imb*_ for the optimized groups (with specific programs for sub-groups based on individual *FV*_*imb*_ values), while the non-optimized group followed a classical program exactly similar for all subjects. All subjects in the three optimized training sub-groups (velocity-deficit, force-deficit, and well-balanced) increased their jumping performance (12.7 ± 5.7% ES = 0.93 ± 0.09, 14.2 ± 7.3% ES = 1.00 ± 0.17, and 7.2 ± 4.5% ES = 0.70 ± 0.36, respectively) with jump height improvement for all subjects, whereas the results were much more variable and unclear in the non-optimized group. This greater change in jump height was associated with a markedly reduced *FV*_*imb*_ for both force-deficit (57.9 ± 34.7% decrease in *FV*_*imb*_) and velocity-deficit (20.1 ± 4.3%) subjects, and unclear or small changes in *P*_*max*_ (−0.40 ± 8.4% and +10.5 ± 5.2%, respectively). An individualized training program specifically based on *FV*_*imb*_ (gap between the actual and optimal F-v profiles of each individual) was more efficient at improving jumping performance (i.e., unloaded squat jump height) than a traditional resistance training common to all subjects regardless of their *FV*_*imb*_. Although improving both *FV*_*imb*_ and *P*_*max*_ has to be considered to improve ballistic performance, the present results showed that reducing *FV*_*imb*_ without even increasing *P*_*max*_ lead to clearly beneficial jump performance changes. Thus, *FV*_*imb*_ could be considered as a potentially useful variable for prescribing optimal resistance training to improve ballistic performance.

## Introduction

Advancements in strength and conditioning methodologies alongside evolution in physical demands of competition in sports such as rugby, football, volleyball, basketball, or athletics, have led to an increased relevance of high-intensity, ballistic actions. Physical performance in these kinds of sports is clearly determined by high levels of force, power, and velocity during ballistic movements such as sprints, changes of direction, or jumps (Cronin and Sleivert, 2005; Cormie et al., 2010).

Ballistic performances, notably jumping, can be defined as the ability to accelerate body mass, both as much as possible and within the shortest time possible (Samozino et al., 2012). From a mechanical point of view, ballistic push-off performance is thus directly related to the net mechanical impulse produced onto the ground (Winter, 2005). The capability to develop a high net impulse during one lower limb push-off has been associated with muscular mechanical power output capabilities (Newton and Kraemer, 1994; Yamauchi and Ishii, 2007; Samozino et al., 2008; Frost et al., 2010; McBride et al., 2010). Numerous studies have highlighted neuromuscular power as the primary variable related to ballistic performance, yet this analysis only provides a partial representation of the athlete's true maximal mechanical capabilities (Cronin and Sleivert, 2005). Specifically, in the recent years, a new paradigm supports the fact that although ballistic performance such as jumping height is largely determined by maximal power output (*P*_*max*_) that lower limbs can generate (Yamauchi and Ishii, 2007), it is also influenced by the individual combination of the underlying force and velocity mechanical outputs, known as force-velocity (F-v) profile (Samozino et al., 2012, 2014; Morin and Samozino, 2016). Thus, the inclusion of F-v relationship and their contribution to ballistic performance may provide a more accurate and integrative mechanical representation of the athlete's maximal capabilities (Samozino et al., 2012), since they encompass the entire force-velocity spectrum, from the theoretical maximal force (*F*_0_) to the theoretical velocity (*v*_0_) capabilities (Morin and Samozino, 2016).

As shown theoretically (Samozino et al., 2008, 2012) and confirmed experimentally (Samozino et al., 2014), there is, for each individual, an optimal F-v profile that maximizes the ballistic performance (e.g., vertical or inclined jumping) and represents the optimal balance between force and velocity qualities for these movements (Samozino et al., 2012, 2014). The relative difference between actual and optimal F-v profiles for a given individual represents the magnitude and the direction of the unfavorable balance between force and velocity qualities (i.e., force-velocity imbalance, *FV*_*imb*_ in %), which makes possible the individual determination of force or velocity deficit. The actual individual F-v profile and *P*_*max*_ can be easily determined from a series of loaded vertical jumps (Samozino et al., 2008, 2014; Jiménez-Reyes et al., 2014, 2016; Giroux et al., 2015, 2016), while the optimal F-v profile can be computed using previously proposed equations based on a biomechanical model (Samozino et al., 2012, 2014). For a given *P*_*max*_, vertical jump performance has been shown to be negatively correlated to *FV*_*imb*_, which supports the importance of considering this individual characteristic in addition to *P*_*max*_ when designing training programs to improve ballistic performance (Samozino et al., 2012, 2014; Morin and Samozino, 2016).

Quantifying *FV*_*imb*_ on an individual basis could therefore help improve the effectiveness of training prescription by adapting it to each athlete's individual needs. In theory, this would lead to improved ballistic performance through an effective shift in the individual actual F-v profile toward the optimal value (*FV*_*imb*_ reduction), and/or an increase in *P*_*max*_ (Samozino et al., 2014). For instance, the individual jumping F-v profile has been shown sensitive to training over different periods of a season in high-level rugby players (de Lacey et al., 2014), and to training history and sport activities of various populations (Vuk et al., 2012; Markovic et al., 2013; Samozino et al., 2014; Giroux et al., 2016). Therefore, we can reasonably expect (personal unpublished pilot observations) that the individual F-v profile would respond to specific training. Moreover, the individual jumping F-v profile has been shown sensitive to the kind of strength training performed (Cormie et al., 2010).

Some traditional training methods have been considered for power improvement, such as: power and ballistic training (e.g., Wilson et al., 1993; Newton et al., 1996; Cormie et al., 2007, 2010; Argus et al., 2011; Markovic et al., 2011; Sheppard et al., 2011; Zaras et al., 2013), heavy-load training (focusing more on strength; e.g., Gorostiaga et al., 1999; Harris et al., 2000; Chelly et al., 2009; Rønnestad et al., 2012, 2016) and combined training (strength-power training; e.g., Wilson et al., 1993; McBride et al., 2002; Kotzamanidis et al., 2005; Cormie et al., 2007, 2010; Smilios et al., 2013; Zaras et al., 2013). This kind of global power training prescription similar for all athletes resulted in contrasting findings as to the effects on jumping performance (e.g., Wilson et al., 1993; Gorostiaga et al., 1999; Harris et al., 2000; McBride et al., 2002; Kotzamanidis et al., 2005; Cormie et al., 2007, 2010; Chelly et al., 2009; Rønnestad et al., 2012, 2016; Smilios et al., 2013; Zaras et al., 2013), likely because of the various levels and F-v characteristics of the populations tested. Indeed, a training program leading to improve *P*_*max*_ while increasing *FV*_*imb*_ could result in a lack of change, or even a decrease in jumping performance. We propose here that tailoring the training prescription to the athlete's individual F-v profile would improve the effectiveness of such training. This innovative approach will be designed as follows.

In the case of a force deficit, training should be aimed to increase *P*_*max*_ while decreasing *FV*_*imb*_, by increasing force capabilities (*F*_0_) as a priority (Samozino et al., 2012). Previous studies clearly shows the effectiveness of strength training aiming at specifically increasing maximal force capabilities (Cormie et al., 2007, 2010; Rønnestad et al., 2012, 2016; Zaras et al., 2013). They have shown improvements in maximal strength parameters (e.g., 1RM, 1RM/BM ratio) through trainings involving the use of high loads (>70% RM), in order to achieve the maximal neuromuscular adaptations, in periods ranging from 6 to 12 weeks. Note that these training-induced adaptations were not systematically associated to ballistic performance improvements.

At the other end of the F-v spectrum, in case of a velocity deficit, training should aim to increase *P*_*max*_ by improving maximal velocity capabilities (i.e., capacity to produce force at very high contraction velocities). It should be oriented toward maximal velocity efforts during high accelerated movements with minimal or null braking phase, for example a throw at the end of a lift and displacing low (<30% RM) or negative loads (Argus et al., 2011; Markovic et al., 2011; Sheppard et al., 2011). The effects of this type of training, commonly referred to as ballistic, can be observed in studies where protocols with a removed deceleration phase during liftings have been shown more effective (Newton et al., 1996; Cormie et al., 2010). These studies and others (Markovic and Jaric, 2007; Argus et al., 2011; Markovic et al., 2011, 2013; Sheppard et al., 2011) show how employing loads lower than body mass (referred to as negative loads) may result in a training-induced shift in force-time curves and force-velocity relationships toward more velocity-related capabilities (Djuric et al., 2016).

Finally, in case of a low deficit (i.e., actual F-v profile close to the computed optimal profile), the training program should target a balanced combination of force, velocity, and power in order to shift the entire F-v relationship to the right, and so to increase *P*_*max*_ as a priority (Wilson et al., 1993; Harris et al., 2000; McBride et al., 2002; Kotzamanidis et al., 2005; e.g., Cormie et al., 2007; de Villarreal et al., 2011) while maintaining the F-v profile close to the optimal value (and thus *FV*_*imb*_ close to 0%). The effects of studies aiming to both increase maximal power and shift the entire F-v curve show how combining a wide range of loads (heavy, optimal, and ballistic loads) is an appropriate stimulus (Harris et al., 2000; McBride et al., 2002; Kotzamanidis et al., 2005; Cormie et al., 2007).

In light of the three latter paragraphs showing the sensitivity of the F-v profile to specific training programs can result in either maximal force or velocity capabilities improvements, it is reasonable to hypothesize that specific resistance training programs can be designed on an individual basis to both reduce *FV*_*imb*_ (i.e., to increase preferably the *F*_0_ or *v*_0_ component of an individual's F-v profile and shift it toward his optimal profile) and increase *P*_*max*_ (in case of well-balanced F-v profile). This is what we will term “optimized training” or “individualized training based on *FV*_*imb*_” in the present study.

Theory (Samozino et al., 2012, 2014) and case studies (Morin and Samozino, 2016) show that, *ceteris paribus*, such a training approach could result in a decreased *FV*_*imb*_ and in turn, in an improved jumping performance. To date no direct controlled experiment has been performed to confirm the effectiveness of this optimized training approach.

The aim of this study was to experimentally test the hypothesis that an individualized training program based on the imbalance in the F-v profile of each individual is more effective in improving jump performance than a traditional resistance training common to all subjects and designed without taking account of individual differences in the initial F-v profiles and imbalances. We also hypothesized that in such a case, the improved vertical jump performance would result from both a shift in the F-v profile toward the optimal and an increase in *P*_*max*_, while the “non-optimized” group would only increase *P*_*max*_. Note that vertical jump height was used here as the index of performance since it represents the archetype of ballistic movements.

## Materials and methods

### Subjects

Eighty-four trained athletes (age = 23.1 ± 4.4 year, body mass = 75.5 ± 8.5 kg, stature = 1.79 ± 0.046 m) gave their written informed consent to participate in this study, which was approved by the local ethical committee of the Catholic University of San Antonio (Murcia) in agreement with the Declaration of Helsinki. All subjects were semi-professional soccer and rugby players. All athletes had a strength-training background ranging from 1 to more than 3 years, were highly trained (average weekly training volume of 12 h at the time of the study), and familiar with the testing procedures.

### Testing procedure and data processing

#### F-v relationships of lower limb neuromuscular system in SJ

To determine individual F-v relationships, each subject performed vertical maximal SJ without loads and against five to eight extra loads ranging from 17 to 87 kg in a randomized order. The test was performed on a Smith machine (Multipower Fitness Line, Peroga, Spain) that allows a smooth vertical displacement of the bar along a fixed vertical path. Before each SJ condition with no additional load, participants were instructed to stand up straight and still on the center of the jumping area. They kept their arms on their hips for jumps without load and on the bar for loaded jumps, this hand position remaining the same during the entire movement. Subjects were asked to maintain their individual starting position (~90° knee angle) for about 2 s and then apply force as fast as possible and jump for maximum height. Countermovement was verbally forbidden and carefully checked. If all these requirements were not met, the trial was repeated. Two valid trials were performed with each load with 2 min of recovery between trials and 4–5 min between loads condition.

Mean mechanical parameters were calculated for each loading condition using Samozino's method (Samozino et al., 2008), based on Newton's second law of motion. This method establishes that mean force (*F*), velocity (*v*), and power (*P*) can be calculated during a vertical jump from jump height and squat jump positions measurement. Jump height was obtained using an OptoJump optical measurement system (Microgate, Bolzano, Italy). Force, velocity and power were calculated using three equations considering only simple input variables: body mass, jump height and push-off distance. The latter corresponds to the distance covered by the center of mass during push-off, i.e., to the extension range of lower limbs from the starting position to take- off (Samozino et al., 2008), and was a priori measured for each subject by the difference between the extended lower limb length (iliac crest to toes with plantar flexed ankle) and the height in the individual standardized starting position (iliac crest to ground vertical distance).

F-v relationships were determined using the best trials of each loading condition and least squares linear regressions. F-v curves were extrapolated to obtain *F*_0_ (then normalized to body mass) and *v*_0_, which respectively correspond to the intercepts of the F-v curve with the force and velocity axis. The F-v profile, that is the slope of the F-v linear relationship was then computed from *F*_0_ and *v*_0_ according to Samozino et al. (2012). Values of *P*_*max*_ (normalized to body mass) were determined as: *P*_*max*_ = *F*_0_·*v*_0_/4 (Samozino et al., 2012, 2014). From *P*_*max*_ and hpo values, there is an individual theoretical optimal F-v profile (normalized to body mass, in N.s.kg^−1^.m^−1^) maximizing vertical jumping performance that was computed for each subject using equations proposed by Samozino et al. (2012). The F-v imbalance (*FV*_*imb*_, in %), was then individually computed as recently proposed by Samozino et al. (2014):

$$Fv_{imb}=100.\;|1-\frac{S_{Fv}}{S_{Fv}\;opt}|$$

A *FV*_*imb*_ value around 0% indicates a F-v proifile equal to 100% of the optimal proifile (perfect balance between force and velocity qualities), whereas a F-v profile value higher or lower than the optimal indicates a profile too oriented toward force or velocity capabilities, respectively.

### Experimental design

The present study used a longitudinal pre-post design with testing sessions separated by 9 weeks. All tests were conducted at the same time of day, from 17:00 to 21:00. Each subject underwent anthropometric assessment and performed loaded squat jumps (SJ) to determine the individual force-velocity (F-v) relationships, *P*_*max*_ values and F-v imbalance (computations are detailed in the next section). *FV*_*imb*_ was then used as the reference to assign participants to different training intervention groups and sub-groups. Since the main line of the approach tested is that performance improvement would result from increasing *P*_*max*_ and/or decreasing *FV*_*imb*_ (Morin and Samozino, 2016), and given our main hypothesis, *FV*_*imb*_ was the criterion used for designing an individualized training program in this study.

After initial testing of their individual F-v properties, participants were assigned to optimized training group [force deficit (FD) sub-group (*n* = 22; body mass = 72.2 ± 8.3 kg, stature = 1.78 ± 0.062 m], velocity deficit (VD) sub-group (*n* = 18; body mass = 80.6 ± 9.6 kg, stature = 1.811 ± 0.042 m), well-balanced (WB) sub-group (*n* = 6; body mass = 75.6 ± 4.9 kg, stature = 1.783 ± 0.049 m), a non-optimized training group (NO, *n* = 18; body mass = 77.0 ± 6.6 kg, stature = 1.791 ± 0.033 m), or a control group (CG, *n* = 20; body mass = 73.0 ± 7.9 kg, stature = 1.785 ± 0.032 m). The training program was adjusted to the needs of participants in the optimized group according to the *FV*_*imb*_. As a consequence, the training program was slightly different regarding loading but similar in volume among groups, and all subjects were familiar with the exercises used. The training intervention was performed at the middle of the competitive season for all participants.

During the 9 weeks of training, the FD sub-group performed mainly force-oriented (very high loads) training, while the VD sub-group performed velocity-oriented (ballistic, very high velocity of limbs extension) training. The WB sub-group followed a training program covering the entire force-velocity spectrum in equal proportions: heavy loads, power, and ballistic training. All the subjects of the NO training group followed the latter kind of training, independently from each subject's *FV*_*imb*_. NO group was composed by 18 subjects with different initial F-v profile and *FV*_*imb*_ before the training intervention (10 with force deficit and 8 with velocity deficit), with the purpose to match the distribution of the optimized groups as much as possible. Finally, the CG maintained their normal level of activity throughout the duration of the study without performing any kind of strength training. For each subject, jumping F-v profile and performance were tested exactly 1 week before the first training session (pre-training), and again 1 week after the completion of the 9-week training (post-training). The training intervention for all groups was organized following recommendations from the literature: more than three sets/session (Rhea et al., 2002) and a frequency of 2–3 sessions/week for strength (Rhea et al., 2003; Peterson et al., 2004), including plyometrics (Markovic, 2007). All intervention groups performed 18 sets/week (details in Tables 1, 2), which is similar to previous research (Harris et al., 2000; McBride et al., 2002; Cormie et al., 2007, 2010; Argus et al., 2011; Markovic et al., 2011; Sheppard et al., 2011). The training dose required to develop strength is generally described as high frequency (3–5 weekly sessions per muscle group), moderate volume (3–6 sets × 2–6 repetitions × load mass), and high intensity (85–100% one-repetition maximum (1RM) and non-ballistic nature of strength training exercises; while power differs mainly in the intensity (20–70% 1RM) and high movement velocity (i.e., explosive-ballistic; Baechle and Earle, 2000; Fleck and Kraemer, 2004).

**Table 1:** **Force-velocity imbalance categories, thresholds, and associated resistance training load ratios**.

***FV_*imb*_* categories**	**F-v profile in % of optimal thresholds (%)**	Training loads ratio^*^
High force deficit	<60	3 Strength
		2 Strength-power
		1 Power
Low force deficit	60–90	2 Strength
		2 Strength-power
		2 Power
Well-balanced	>90–110	1 Strength
		1 Strength-power
		2 Power
		1 Power-speed
		1 Speed
Low velocity deficit	>110–140	2 Speed
		2 Power-speed
		2 Power
High velocity deficit	>140	3 Speed
		2 Power-speed
		1 Power

*FV_imb_, F-v imbalance*.

* *Ratio based on six exercises/wk, three sets/exercise and 18 sets/wk*.

**Table 2:** **Loading target for the F-v spectrum and exercises and training loads for each exercise**.

**Loading focus/target**	**Exercises**	**Training loads**
Strength	Back squat	80–90% 1RM
	Leg press	90–95% 1RM
	Deadlift	90–95% 1RM
Strength-power	Clean pull	80% 1RM
	Deadlift	80% 1RM
	SJ	>70% of BW
	CMJ	>80% of BW
Power	SJ	20–30% of BW
	CMJ	35–45% of BW
	Single leg SJ	BW
	Single leg CMJ	10% of BW
	Clean pull jump	65% 1RM
Power-speed	Depth jumps	
	SJ	BW
	CMJ	10% of BW
	Maximal Vertical Box Jump	
Speed	Maximal Roller Push-off	<BW
	CMJ with arms	BW

*RM, repetition maximum; SJ, Squat Jump; BW, body weight; CMJ, Countermovement Jump*.

### Training intervention

Due to the pilot feature of this study, we designed straight-forward and simple training programs in the context of a first attempt to test our hypotheses. All training programs involved two sessions per week, each separated by 48 h of recovery. Subjects refrained from any additional lower body resistance training outside the experimental training throughout the course of the study. Their competitive activities and sport-specific training was maintained.

Considering the aforementioned elements on the specificity of training to improve the specific components of maximal force or velocity parts of the F-v spectrum (e.g., Wilson et al., 1993; Newton et al., 1996; Harris et al., 2000; McBride et al., 2002; Cormie et al., 2007, 2010; Argus et al., 2011; Markovic et al., 2011; Sheppard et al., 2011; Rønnestad et al., 2012, 2016; Zaras et al., 2013), the FD and VD training groups were established according to individuals' *FV*_*imb*_. For each one of these sub-groups, we considered not only the type of deficit (either in force or in velocity), but also its magnitude. Therefore, in each sub-group, the training program was established according to specific *FV*_*imb*_ thresholds, as detailed in (Table 1).

According to previous findings showing improvements in maximal strength, power and ballistic performance after specific training (e.g., Cormie et al., 2007, 2010), the individualized training programs proposed here included maximal efforts and were mainly designed by setting the loads to vary the movement velocity, and in turn to target the different parts of the F-v curve. For example, “Strength” exercises used high loads ~ *F*_0_ moved at low v such as >80% of one repetition maximum in back squat whereas “Speed” exercises used *F* of ~body mass moved at high *v*. The high *v* was greater than a squat jump, using the stretch-shortening cycle (e.g., CMJ) or assisted/low resistance push-offs (e.g., band assisted SJ or horizontal assisted roller).

### Statistical analysis

All data are presented as mean ± *SD*. In order to clearly assess the practical meaning of the results, data were analyzed using the magnitude-based inference approach (Hopkins et al., 2009).

Within-group difference in pre and post-training jump height, F-v profile in (%) of Optimal F-v, *F*_0_, and *v*_0_ were assessed using standardized effect size (ES). Between-group (optimized group vs. non-optimized group) differences in pre and post-training jump height and F-v profile in (%) of Optimal F-v were also assessed using standardized ES. The magnitude of the within-group and between-group changes was interpreted by using values of trivial (<0.20), small (0.20 to < 0.60), moderate (0.60 to < 1.20), large (1.20 to < 2.00), and extremely large of the between-athlete variation at pre (i.e., smallest worthwhile change, SWC).

The probability that these differences actually exist was then assessed via magnitude-based qualitative inferences (Batterham and Hopkins, 2006). Qualitative inferences were based on quantitative chances of benefit outlined in Hopkins et al. (2009). Clinical chances are percentage chances that an observed effect is clinically positive/trivial/negative e.g., (40/40/20%) means an effect has 40% of chances to be positive, 40% to be trivial, and 20% to be negative. Probabilities that differences were higher than, lower than, or similar to the smallest worthwhile difference were evaluated qualitatively as possibly, 25–74.9%; likely, 75–94.9%, very likely, 95–99.5%; and most (extremely) likely, >99.5%.

Since the findings of present study could be used for athletes considered in isolation, individual analyses were performed to quantify for each variables and each group the number of responders and no responders. Monitoring progression of an athlete with performance requires taking into account the magnitude of the SWC in performance and the uncertainty or noise in the test result (Hopkins, 2004), SWC being computed as one-fifth of the between-athlete standard deviation (a standardized or Cohen effect size of 0.20, Hopkins, 2004). Subjects were then considered as harmful (individual change < −1 SWC), trivial (from −1 SWC to +1 SWC) or beneficial (+1 SWC) responders.

## Results

Mean ± *SD* values for all performance and mechanical variables pre and post training intervention are shown for all groups and sub-groups in Table 3, along with within-group changes qualitative inferences.

**Table 3:** **Changes in variables associated to Force-velocity profile in different groups**.

	**Pre**	**Post**	**Post-pre**				**Individual response**
	**$\bar{\text{x}}$ ± *SD***	**$\bar{\text{x}}$ ± *SD***	**%Δ ± *SD***	**ES; ± 90% CL**	**Inference and probability**		**Harmful/Trivial/Beneficial**
**F-v (%) OPTIMAL F-v**							
Force deficit	45.1 ± 14.3	68.8 ± 17.7	57.9 ± 34.7	1.60 ± 0.26	Large +ive	most likely	1 – 0 – 21
Velocity deficit	130 ± 11.5	103 ± 6.3	−20.1 ± 4.3	−2.20 ± 0.26	Ext. Large –ive	most likely	0 – 0 – 18
Well-balanced	101 ± 7.0	100 ± 1.4	−0.50 ± 6.7	−0.11 ± 0.20	Trivial	unclear	3 – 1 – 2
Non-optimized	88.6 ± 38.1	81.8 ± 34.7	−5.54 ± 17.6	−0.17 ± 0.15	Trivial	possibily	7 – 3 – 8
Control	77.9 ± 33.1	79.3 ± 34.5	1.91 ± 17.1	0.01 ± 0.08	Trivial	most likely	1 – 14 – 5
***P**_***max***_* **(W**·**kg**^−1^**)**							
Force deficit	30.7 ± 5.6	30.5 ± 5.8	−0.40 ± 8.4	−0.04 ± 0.17	Trivial	likely	7 – 12 – 3
Velocity deficit	24.2 ± 4.8	26.6 ± 5.0	10.5 ± 5.2	0.48 ± 0.08	Small +ive	most likely	0 – 1 – 17
Well-balanced	23.9 ± 2.2	25.2 ± 2.2	5.53 ± 4.5	0.50 ± 0.33	Small +ive	likely	0 – 1 – 5
Non-optimized	23.5 ± 3.5	24.0 ± 3.3	2.42 ± 6.1	0.13 ± 0.16	Trivial	likely	5 – 3 – 10
Control	23.2 ± 2.5	23.4 ± 5.6	0.61 ± 15.7	0.09 ± 0.58	Trivial	unclear	7 – 11 – 2
**F**_0_ **(N**·**kg**^−1^**)**							
Force deficit	29.1 ± 4.1	35.9 ± 4.2	24.0 ± 10.8	1.60 ± 0.17	Large +ive	most likely	0 – 2 – 20
Velocity deficit	43.4 ± 6.1	40.6 ± 5.2	−6.16 ± 3.3	−0.43 ± 0.10	Small -ive	most likely	17 – 1 – 0
Well-balanced	38.5 ± 1.5	39.1 ± 1.9	1.76 ± 3.5	0.38 ± 0.64	Small +ive	unclear	2 – 1 – 3
Non-optimized	34.1 ± 7.4	33.2 ± 7.1	−2.32 ± 6.8	−0.12 ± 0.13	Trivial	likely	9 – 6 – 3
Control	31.9 ± 6.8	31.9 ± 7.0	0.21 ± 3.8	0.00 ± 0.06	Trivial	most likely	2 – 17 – 1
**v**_0_ **(m**·**s**^−1^**)**							
Force deficit	4.29 ± 0.93	3.44 ± 0.78	−18.9 ± 11.8	−0.88 ± 0.24	Moderate -ive	most likely	21 – 0 – 1
Velocity deficit	2.21 ± 0.16	2.60 ± 0.17	17.9 ± 4.2	2.73 ± 0.21	Ext. Large +ive	most likely	0 – 0 – 18
Well-balanced	2.48 ± 0.17	2.57 ± 0.11	3.70 ± 4.1	0.44 ± 0.41	Small +ive	likely	1 – 1 – 4
Non-optimized	2.88 ± 0.72	3.00 ± 0.67	5.57 ± 11.9	0.17 ± 0.21	Trivial	possibly	5 – 3 – 10
Control	3.05 ± 0.82	3.10 ± 1.25	1.18 ± 21.2	0.06 ± 0.36	Trivial	unclear	3 – 16 – 1
**JUMP HEIGHT (m)**							
Force deficit	0.323 ± 0.04	0.367 ± 0.04	14.2 ± 7.3	1.00 ± 0.17	Moderate +ive	most likely	0 – 0 – 22
Velocity deficit	0.319 ± 0.06	0.357 ± 0.05	12.7 ± 5.7	0.93 ± 0.09	Moderate +ive	most likely	0 – 0 – 18
Well-balanced	0.315 ± 0.03	0.338 ± 0.03	7.22 ± 4.5	0.70 ± 0.36	Moderate +ive	very likely	0 – 0 – 6
Non-optimized	0.305 ± 0.04	0.312 ± 0.04	2.33 ± 4.7	0.14 ± 0.13	Trivial	likely	1 – 10 – 7
Control	0.292 ± 0.04	0.288 ± 0.04	−1.43 ± 3.3	−0.09 ± 0.10	Trivial	very likely	9 – 10 – 1

*Values are mean ± standard deviation, percent change ± standard deviation and standardized effect size; ±90% confidence limits. $\bar{x}$, mean; SD, standard deviation, %Δ, percent change; ES, effect size; 90% CL, 90% confidence limits; Ext, extremely; +ive, positive effect; -ive, negative effect; P_max_, maximal power output; W, watt; kg, kilogramme; F_0_, theoretical maximal force; N, newton; v_0_, theoretical maximal velocity; m, metre; s, second. Qualitative inferences are trivial (<0.20), small (0.20 to <0.60), moderate (0.60 to <1.20), large (1.20 to <2.00) and extremely large moderate (>2.00): possibly, 25 to <75; likely, 75 to <95%; very likely, 95 to <99.5%; most likely, >99.5. Positive, neutral and negative descriptors qualitatively describe the change between post and pre values and its importance relative to the specific variable*.

**Figure 1:**
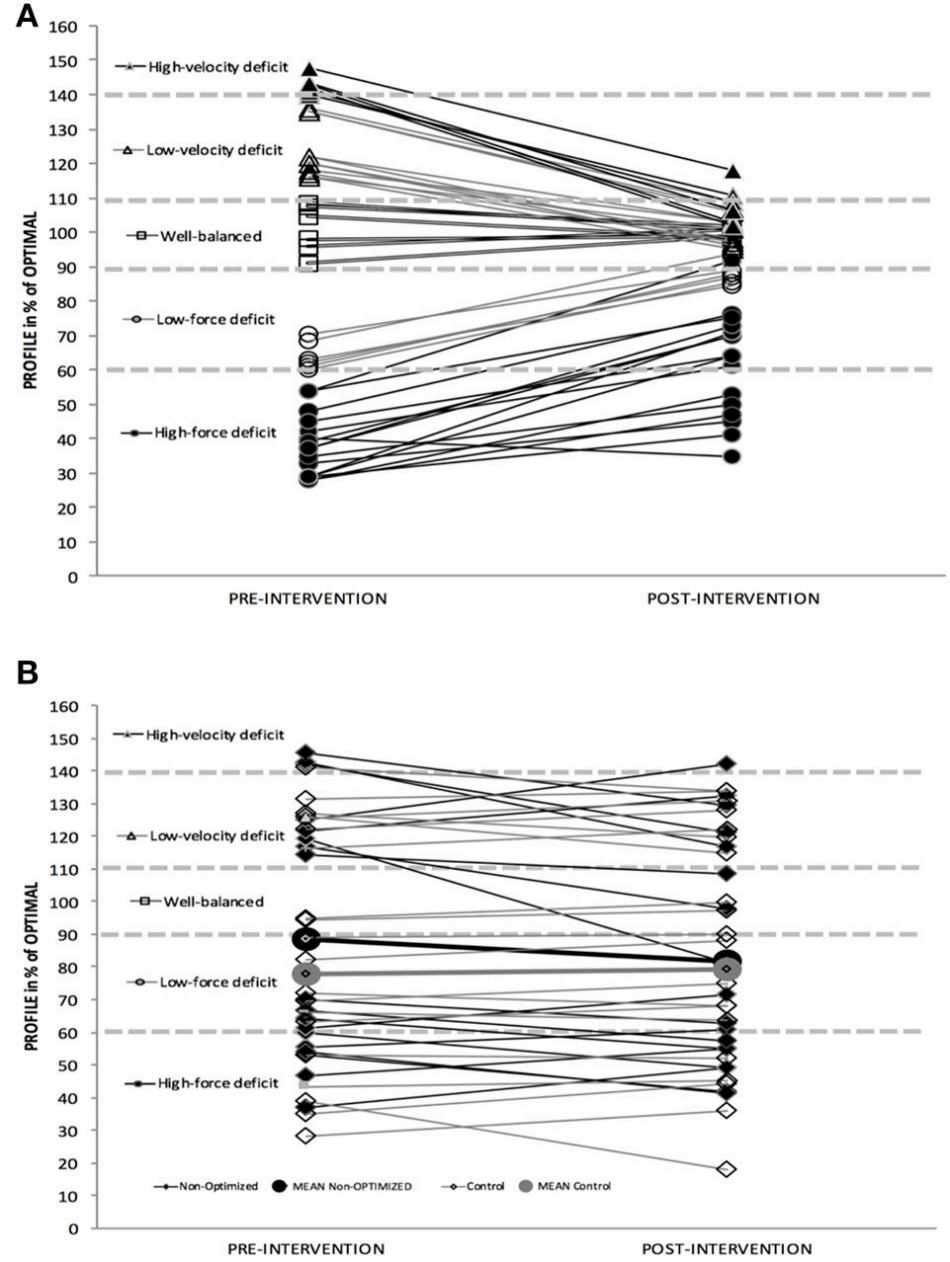
**(A)** Individual Pre-Post changes in F-v profile for Optimized Group and sub-groups. **(B)** Individual Pre-Post changes in F-v profile for Non-Optimized and Control Group.

**Figure 2:**
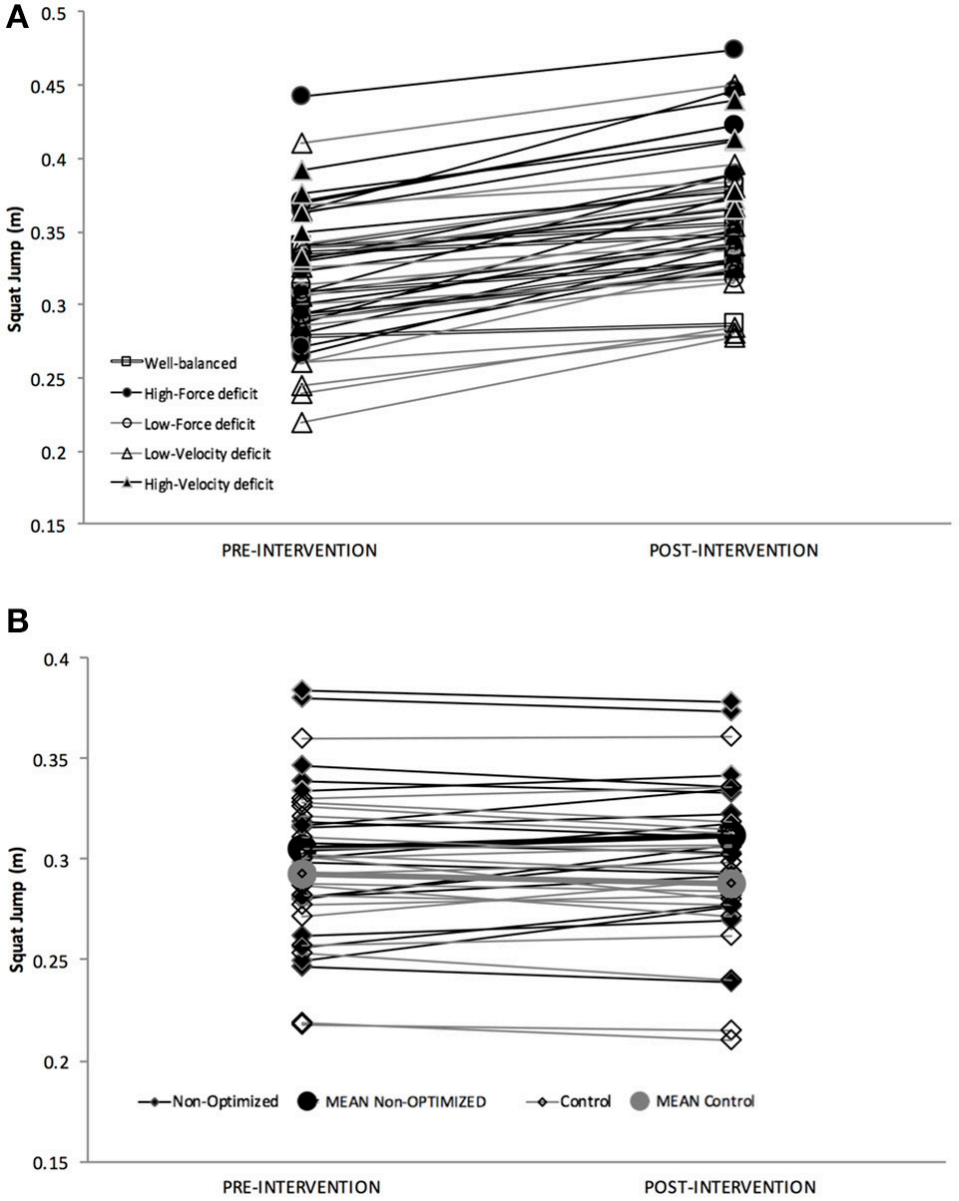
**(A)** Individual Pre-Post changes in vertical jump height for Optimized Group and sub-groups. **(B)** Individual Pre-Post changes in F-v profile for Non-Optimized and Control Group.

FD and VD sub-groups showed large and extremely large changes in *FV*_*imb*_, respectively, whereas this change was unclear for the WB group, who showed a small increase in *P*_*max*_ (Table 3). Contrastingly, the changes for the “non-optimized” group were overall likely trivial, with a very high inter-subject variability (Table 3; Figures 1, 2). The most important improvements in jump performance were observed in the intervention groups (+7.2 to +14.2% on average, very likely to most likely very large effects vs. trivial change of +2.3% for the non-optimized and control group).

An individual approach of the study outcomes shows that in the optimized group (including FD, VD, and WB sub-groups) all the 46 subjects improved their jump height, and all of them but one reduced their *FV*_*imb*_ (Table 3). For the non-optimized group, only 10 subjects out of 18 improved jump height and 11 out of 18 reduced their *FV*_*imb*_. Finally, in the control group, only 8 subjects out of 20 subjects improved jump height and 12 out of 20 reduced their *FV*_*imb*_.

In the optimized group (including FD, VD, WB sub-groups), all the subjects were beneficial responders in jump height, and all of them but one in *FV*_*imb*_ (Table 3). Contrastingly, 7 subjects out of 18 of the non-optimized group were beneficial responders for jump height (8 out of 18 for *FV*_*imb*_) and 1 out of 20 in control group (5 out of 20 in *FV*_*imb*_, Table 3).

The results referring to between-groups differences showed almost certain large (ES = 1.21–100/0/0) and moderate (ES = 0.73 100/0/0) differences in *FV*_*imb*_ and jump height, respectively, in favor of optimized group vs. non-optimized group, which highlight the findings in our study.

As shown in Table 3, the changes for the non-optimized and control groups were much more variable and the overall magnitude less clear.

## Discussion

The aim of this study was to experimentally test the hypothesis that an individualized training program based on the force-velocity imbalance of each subject was more effective at improving jump performance than a traditional resistance training common to all subjects (designed without taking account of individual differences in the initial F-v profiles and imbalances). Our main hypothesis was that this individualized and optimized training would result in a decreased imbalance for each individual, and in turn a greater vertical jump performance. The main findings of this study validate our initial hypothesis: (i) an optimized and individualized training program specifically addressing the *FV*_*imb*_ is more efficient for improving jumping performance (all subjects showed improvement in jump height) than a traditional resistance training common to all subjects (10 subjects out of 18 improved), regardless of their force-velocity imbalance and optimal force-velocity profile, (ii) for subjects with an initial substantial *FV*_*imb*_ (i.e., force or a velocity deficit) this higher jump performance was associated with the sensitivity of the force-velocity profile to the specific training program tailored to the athlete's individual needs, which has lead here to a reduced F-v imbalance with no change in *P*_*max*_.

It is important to highlight that jump height improvements were greater, and for a larger number of subjects, with a training based on the F-v approach and *FV*_*imb*_ as opposed to traditional training. Although this is not a common practice in the strength and conditioning field, its application could be of high interest since the optimal F–v profile, which can be accurately determined, depends on individual characteristics (limb extension, *P*_*max*_ and different loaded squat jumps; Samozino et al., 2012). Thus it could be a very easy to design a training program with the focus on increasing *P*_*max*_ and/or decreasing *FV*_*imb*_ (Morin and Samozino, 2016).

The main novelty of our results was that in order to improve jump performance, not only should *P*_*max*_ be considered, but also the individual force-velocity profile. Indeed, changes in *P*_*max*_ were trivial or small in the FD and VD optimized sub-groups, whereas changes in *F*_0_ and *v*_0_ were large and extremely large. Contrastingly, the “one-size-fits-all” program with the non-optimized group might not be an efficient training stimulus for a group of individuals with different *FV*_*imb*_, thus different training needs. Despite some inevitable limitations that will be addressed at the end of this discussion, the novelty of the present experimental study and the clear results obtained may bring valuable additional knowledge and potential applications to sport training practice toward a more individualized, specific, and effective training monitoring and periodization.

Several studies have shown some positive effects of strength training on improving vertical jump performance, although with contradictory results, and inconsistancies in the training prescription (e.g., heavy loads for all subjects; Gorostiaga et al., 1999; Harris et al., 2000; McBride et al., 2002; Cormie et al., 2010; de Villarreal et al., 2011; Losnegard et al., 2011; Rønnestad et al., 2012, 2016), while other studies considered also light loads (e.g., Wilson et al., 1993; McBride et al., 2002; Cormie et al., 2007; Zaras et al., 2013), or combined strength training (e.g., Wilson et al., 1993; Toji et al., 1997; Harris et al., 2000; McBride et al., 2002; Kotzamanidis et al., 2005; de Villarreal et al., 2011). Two common features of these studies were that the same training program was prescribed to all subjects and the great variability in performance response to training (Wilson et al., 1993; Gorostiaga et al., 1999; Harris et al., 2000; McBride et al., 2002; Kotzamanidis et al., 2005; Cormie et al., 2007, 2010; Chelly et al., 2009; de Villarreal et al., 2011; Losnegard et al., 2011; Rønnestad et al., 2012, 2016; Smilios et al., 2013). In the present protocol, all the subjects tested were sub-divided into specific sub-groups and then assigned to a group-specific training program, which led to a more appropriate training program and more efficient and less variable results in terms of jump performance improvement compared to the aforementioned studies. Each of these groups will be discussed separately.

### Force-deficit sub-group

For the FD sub-group, the specific heavy-load program resulted in moderate to large increases in *F*_0_ (+24 ± 10.8% on average; ES = 1.60 ± 0.23), *FV*_*imb*_ reduction for all subjects but one (−57.9 ± 34.7%; ES = 1.60 ± 0.26) and jump height (+14.2 ± 7.3%; ES = 1.00 ± 0.17). Individual analysis showed that all subjects improved jump height and all but one reduced *FV*_*imb*_, which would support the great effectiveness of this kind of training approach. Thus, as hypothesized, the selected exercises for this group were likely effective for specifically shifting F-v profile in accordance with initial *FV*_*imb*_ showing a force-deficit (Table 3; Figures 1, 2). These findings are in agreement with other studies showing high-load training specificity (Wilson et al., 1993; Harris et al., 2000; McBride et al., 2002; Cormie et al., 2010).

The large increase in *F*_0_ in the FD sub-group was associated to an important increase in jump height since vertical jumping is in the most force-dependent plane for a ballistic movement as it encounters the maximal magnitude of negative gravitational acceleration (Minetti, 2002; Samozino et al., 2012) meaning an increase in *F*_0_ would influence jump height more than an increase in *v*_0_ (Samozino et al., 2010). In line with this, the FD group had “high” or “low/moderate” force deficits and thus used mainly heavy load resistance training exercises (Tables 1, 2) which should have theoretically increased jump height and principally via an increase in *F*_0_ (Harris et al., 2000; McBride et al., 2002; Cormie et al., 2010; Losnegard et al., 2011; Rønnestad et al., 2012, 2016).

The increase in *F*_0_ is observed here in parallel with a decrease in *v*_0_, even if no interrelationships can be supported between these two qualities, except the fact that when one of these qualities is trained, the other is not. So, in present study, the maximal strength improvement (*F*_0_) is not associated with the same kind of increase in *P*_*max*_, which would have been the case if subjects had kept their *v*_0_ value similar. Consequently, the performance improvement can be only attributed to *FV*_*imb*_ reduction, and not to an increased *P*_*max*_, which is greater support for the interest of considering *FV*_*imb*_ in strength training focusing on improving ballistic performance.

The possible explanatory mechanisms for these changes in maximal force after the optimized training may include an increased neural drive and enhanced intermuscular coordination (McBride et al., 2002; Cormie et al., 2010), rate of motor unit recruitment (size principle), and the activation of type II muscle fibers and subsequent improvement in maximal strength capabilities (Cormie et al., 2011). These changes may also be associated with effective changes in synchronization of action potentials and antagonist co-activations leading to an improvement in dynamic force and power production (Folland and Williams, 2007).

As discussed in the limitations, the duration of the training intervention induced a shift in F-v profile (*FV*_*imb*_ reduced by more than one half), although some additional weeks of training might have totally removed *FV*_*imb*_ in some subjects, since the time for adjustments at structural level (mainly related to *F*_0_) typically require a longer period (Kenney et al., 2015) beyond the more acute neuromuscular adaptations.

### Velocity-deficit sub-group

Concerning the VD group, the specific training caused moderate to extremely large increases in *v*_0_ (+17.9 ± 4.2%; ES = 2.37 ± 0.21), *FV*_*imb*_ reduction (−20.1 ± 4.3%; ES = 2.20 ± 0.26) and jump height (+12.7 ± 5.7%; ES = 0.93 ± 0.09), with a majority of responders (Table 3; Figures 1, 2). This suggests that the selected specific exercises for this group were effective for shifting F-v profile in accordance with initial *FV*_*imb*_ (Table 3; Figures 1, 2) and improving the maximal velocity end of the F-v relationship. These findings are in agreement with other studies aiming at specifically improving velocity qualities (Newton et al., 1996; Argus et al., 2011; Markovic et al., 2011; Sheppard et al., 2011), supporting the “principle of velocity specificity” as a specific stimulus to promote velocity-specific neural training adaptations (Kanehisa and Miyashita, 1983; Sale, 1988; Newton et al., 1996; Paddon-Jones et al., 2001). Similarly to the FD sub-group, the increase in *v*_0_ in the VD group was observed here in parallel with a decrease in *F*_0_, so following the same interpretation as above, the performance improvement can only be attributed to *FV*_*imb*_ reduction, and not to increased *P*_*max*_. It is almost certain that with both a *FV*_*imb*_ reduction and an increase in *P*_*max*_ (so keeping *F*_0_ at similar level), improvements in performance would have been greater.

**Figure 3:**
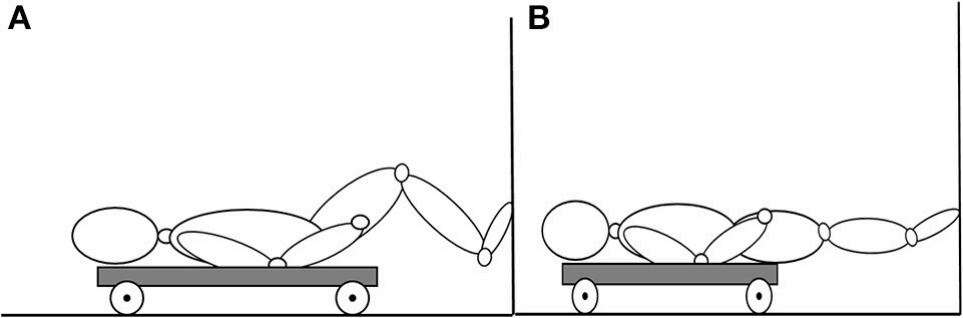
**(A)** Representation of starting position for horizontal push-off exercise. **(B)** Representation of final position for horizontal push-off exercise.

The main exercise used in the VD group was the “horizontal push” (Figure 3) exercise in which there was an almost total elimination of the gravitational component. This could be explained by the fact that power output developed during maximal efforts is less dependent on muscle strength when the exercise does not involve gravity (Minetti, 2002), as it would be the case of a horizontal extension such as our horizontal push exercise. This training exercise likely had a meaningful impact in the extremely large increase in *v*_0_ observed for the VD group.

The main features of this exercise (almost null gravitational component and limited time available for applying force) makes it particularly suitable for the development of explosive muscle contractions (rate of force development and maximal velocity; Tillin and Folland, 2014). This “overspeed” exercise may therefore be considered of great interest for improving velocity capabilities and reducing the associated *FV*_*imb*_ since it suppresses the gravitational constraint (Markovic and Jaric, 2007; Sheppard et al., 2011; Leontijevic et al., 2012; Vuk et al., 2012; Markovic et al., 2013; Pazin et al., 2013; Suzovic et al., 2013) and helps athletes reach lower limbs extensión velocity 20–30% higher than the take-off velocity of a SJ (personal unpublished data; Markovic et al., 2011). As a result, the most representative effect would be to shift the early force-time curve to the left (50–100 first ms; Oliveira et al., 2013, 2016). That said, from a practical standpoint, we think that any exercise involving an external resistance force lower than bodyweight can be effective. Although further studies should investigate whether lighter loads have greater training effects, these results are in line with studies about the influence of training with no gravitational negative loads (Argus et al., 2011; Markovic et al., 2011; Sheppard et al., 2011). Sheppard et al. (2011) found that volleyball players who trained for 5 weeks with assisted jumps, significantly improved jump height compared to subjects who performed regular jump training. They reported that “assisted jumping” was useful at overloading the velocity aspects of jump propulsión, which is similar to what we intended to do with a specific work of horizontal push. Similarly, (Markovic et al., 2011), reported a marked increase in peak lower limb extension velocity resulting from a 7-week jump training with negative loading (assisted jumps), supporting the concept of velocity specificity in resistance training as in previous ballistic power training studies (Sale, 1988; Markovic and Jaric, 2007; Cormie et al., 2010). Finally, Argus et al. (2011) observed similar positive effects on jump height after a 4-week assisted jumps (−20% of body weight) training program. Interestingly, these authors reported the individual changes, which show that 6 of the 9 participants in their study improved jump height under this “velocity-oriented” program. The fact that these subjects were highly trained rugby players (thus very likely with a force-oriented F-v profile, and in turn a velocity deficit) may explain the counterintuitive result showing that most of them improved jump height after an assisted jump training program. We speculate that most of these rugby players needed to correct a velocity deficit to improve jump height, and this may explain why they responded well to the assisted jump program.

Although our aim was not to investigate the neuro-physiological mechanisms underpinning the changes observed in the VD group, these may include a changes in neural activation patterns (i.e., lowered motor unit recruitment thresholds, improved motor unit firing frequency, and possibly synchronization, greater and/or more effective recruitment of fast-twitch muscle fibers) and enhanced intra- and inter-muscular coordination (Sale, 1988; Behm and Sale, 1993; VanCutsem et al., 1998; de Ruiter et al., 2004, 2006).

Another interesting yet secondary result was that in the FD and VD groups, the decrease in *FV*_*imb*_ was due to both an increase in the targeted component of the F-v profile (i.e., *F*_0_ and *v*_0_, respectively), but also a decrease in the opposite component. Subjects in the FD groups showed a large decrease in *v*_0_ after training (−18.9 ± 11.8%) and subjects in the VD group showed a decrease in *F*_0_ (−6.2 ± 3.3%). Although at first sight it may seem that a training program resulting in a decrease of a physical quality is counterproductive, but these changes overall contributed here to the effective shift in the F-v profile toward the optimal value. As previously mentioned, better improvements might have been observed if subjects had both an increase in their weak component (e.g., *F*_0_ for the FD group) and a maintainance of the other component (e.g., *v*_0_ for the FD group), which lead to an optimization of the F-v profile (certainly slightly less fast than here) and an increase in *P*_*max*_, both of them contributing independently to improve performance. The training protocols proposed in this study did not allow subjects presenting a F-v deficit to maintain at a similar level the muscular quality initially considered as a “strength.” The latter requires to keep the training quality considered as a “strength” in addition to focus on reducting the “weakness,” which was not done enough in present protocol. However, the increase in performance obtained here without any large change in *P*_*max*_ well supports the interest to consider *FV*_*imb*_ in training focusing to improve ballistic performance.

### Well-balanced sub-group

Regarding the WB group results, the training program encompassed all the components of the force-velocity spectrum with equal distribution, and resulted in increases in *F*_0_ (+1.8 ± 3.5%; ES = 0.38 ± 0.64) and *v*_0_ (+3.7 ± 4.1%; ES = 0.44 ± 0.41) capabilities, with an overall maintainance of F-v profile (average *FV*_*imb*_ change of −0.5 ± 6.7%; ES = 0.11 ± 0.20). This group showed a moderate jump height improvement (+7.2 ± 4.5%; ES = 0.70 ± 0.36) mainly explained by the small increase in *P*_*max*_. Thus, the training program proposed to this group resulted in an overall shift of the entire F-v relationship to the right: increasing *P*_*max*_ while maintaining a very low *FV*_*imb*_, i.e., F-v profile close to the optimal value. The findings for this group are in agreement with previous studies using mixed resistance training loads (Wilson et al., 1993; Toji et al., 1997; Harris et al., 2000; McBride et al., 2002; Toji and Kaneko, 2004; Kotzamanidis et al., 2005; Cormie et al., 2007; de Villarreal et al., 2011) to increase maximal power output, and in turn ballistic performances.

### Non-optimized group

The “non-optimized” group performed a training program that was similar to the WB group, and similar among subjects, whatever their individual *FV*_*imb*_ and thus training needs. This resulted in a less clear changes in jump height (+2.3% on average ± 4.7%; ES: 0.14 ± 0.13, likely trivial), and substantial inter-individual variability. Furthermore, only 10 subjects out of 18 improved jump height in this group (although only 7 out of 18 were beneficial responders, Table 3) vs. all subjects in the FD, VD and WB sub-groups. This result was associated with variable changes (coefficient of variability of 200% or more) in the F-v mechanical outputs: change in *F*_0_ was −2.3 ± 6.8%; *v*_0_: +5.6 ± 11.9%; *P*_*max*_: +2.4 ± 6.1%; and *FV*_*imb*_: −5.5 ± 17.6%. These results show that when addressed as a group, and not individually, the training program was not effective in reducing each subject's *FV*_*imb*_. On the contrary, this common program induced positive changes for some subjects and negative changes for others, and overall considerable variability in the outcome for both mechanical features of the F-v profile and jump performance (Table 3; Figures 1, 2). Comparatively, in terms of jump height improvement, the number of responders in this non-optimized group (7 out of 18) was much lower than for the optimized sub-groups (all subjects of FD, VD, and WB groups, i.e., 56 out of 56). It is important to note that the NO group was composed of 18 subjects with different initial F-v profile and *FV*_*imb*_ before the training intervention (10 with force deficit and 8 with velocity deficit) to match the distribution of the optimized groups as much as possible. Assigning all subjects of an intervention group to a common program aiming at increasing jumping power and performance is the classical modus operandi (Wilson et al., 1993; Harris et al., 2000; McBride et al., 2002; Toji and Kaneko, 2004; Cormie et al., 2007, 2010), and these studies also showed a great inter-individual variation in response to the resistance training program. This high variability could be interpreted as if the training program does not target the initial *FV*_*imb*_ of each subjects (as in our FD, VD, and WB sub-groups), then subjects might not receive the most effective training prescription on an individual basis, which in turn results in variable outcomes when considering group results. Depending on the interaction between subjects' background and the training program proposed, an experimental group may include responders and non-responders in variable proportions (e.g., Wilson et al., 1993; Gorostiaga et al., 1999; Harris et al., 2000; McBride et al., 2002; Kotzamanidis et al., 2005; Cormie et al., 2007, 2010; Chelly et al., 2009; Losnegard et al., 2011; de Villarreal et al., 2011; Rønnestad et al., 2012, 2016; Smilios et al., 2013). This has not been controlled for in previous studies, which we think is a likely explanation for the high variability observed in the outcome of most studies investigating the effects of strength training on jump height. Thus, we suggest that the individual F-v profile and the *FV*_*imb*_ are taken into account as a starting point for prescribing specific loads and training program for a more effective training to improve jump performance (Tillin and Folland, 2014; Morin and Samozino, 2016).

## Limitations

Although this is the first study to tailor the strength training program to the individual characteristics of the F-v profile, it has limitations that should be discussed. First, our subject recruitment led to a WB sub-group of only six subjects while FD and VD sub-groups included 22 and 18 subjects, respectively. Our personal experience (unpublished data) with hundredths of athletes in various sports show that well-balanced F-v profile (i.e., *FV*_*imb*_ close to 0%) are rare compared to subjects with a F-v deficit (be it a force or a velocity deficit). However, the results for these six individuals confirmed our hypothesis that a well-balanced training would maintain their *FV*_*imb*_ at low values, while increasing their *P*_*max*_, and in turn their jump height. The second and main limitation is that the fixed training duration of 9 weeks for all subjects could be considered as not ideal. We think that, just as the content of the training program, its duration should also have been set on an individual basis. The duration of the program should have been the duration necessary for each individual to reach a *FV*_*imb*_ close to 0. As shown in Figure 1 and Table 3, this 9-week duration was close to optimal for most of the VD sub-group subjects, but not long enough for most FD sub-group subjects. We speculate that should the training duration have also been individualized, the results of the intervention would have been even better. We admit that, as explained in the methods, this 9-week duration has been set under the influence of previous studies, and we hope for further studies to show an even more efficient optimized training should individualize both the training content and the training duration, so that each subject reduces his *FV*_*imb*_ and/or increases his *P*_*max*_. This is, to our opinion, one advantage of this approach: it allows a dynamic adaptation to each individual's response to training, both in terms of training content and timing. Furthermore, as we observed for only 2 subjects in this study (mid-program assessment of F-v profile, data not shown), should subjects adapt faster than others and change sub-group (e.g., from high force deficit to low force deficit) within the pre-set training period, intermediate assessments may easily allow to finely tune the training program and adapt it to the response kinetics of each individual. Finally, our study is focused on vertical F-v profile and jump performance only, which is the simplest and most representative movement for ballistic performances. An interesting point would be to test whether a more optimal jumping F-v profile would be associated with improved performance in other maximal effort contexts such as sprint cycling or running. The latter is a major physical component of performance in many sports, and many studies focused on the transfer between lower limbs strength training and sprint running performance (Cronin and Sleivert, 2005; Seitz et al., 2014; Contreras et al., 2016). This would be a complementary step for a better understanding of F-v profile based training for new insights in strength and conditioning practice. Finally, it is worth highlighting that when we are referring to optimized training and performance improvement, we mean ballistic performances performed at body mass without resistance (i.e., when the aim is to maximally accelerate his own mass, and in particular vertical jumping). We chose vertical jump performance in the present study since it is the simplest task which well represents ballistic movements and it is a key direct or indirect physical performance variable in many sport activities.

## Conclusion

An optimized and individualized training program specifically addressing the force-velocity imbalance is more efficient at improving jumping performance than a traditional resistance training common to all subjects regardless of their force-velocity imbalance and optimal force-velocity profile. *FV*_*imb*_ could therefore be considered as a potentially useful variable for prescribing optimal resistance training to improve ballistic (e.g., jumping) performance. As discussed recently (Morin and Samozino, 2016), this force-velocity approach may help improve the training practice for performance in explosive push-off actions like jumping, through a more efficient monitoring and understanding of the individual determinants of athletic performance. The experimental results obtained in the present study confirmed the theoretical principles of the optimized training approach (Samozino et al., 2014; Morin and Samozino, 2016) that jump performance depends not only on maximal power output, but on an optimal force-velocity profile.

## Author contributions

Conceived and designed the experiments: PJ, PS, MB, JM. Performed experiments: PJ. Analyzed data: PJ, PS, MB, JM. Interpreted results of research: PJ, PS, MB, JM. Drafted manuscript and prepared tables/figures: PJ, JM. Edited, critically revised paper, and approved final version of manuscript: PJ, PS, MB, JM.

### Conflict of interest statement

The authors declare that the research was conducted in the absence of any commercial or financial relationships that could be construed as a potential conflict of interest.
